# Point-of-care application of diaphragmatic ultrasonography in the emergency department for the prediction of development of respiratory failure in community-acquired pneumonia: A pilot study

**DOI:** 10.3389/fmed.2022.960847

**Published:** 2022-08-17

**Authors:** Sheng-En Chu, Jian-Xun Lu, Shi-Chuan Chang, Kuang-Hung Hsu, Zhong Ning Leonard Goh, Chen-Ken Seak, Joanna Chen-Yeen Seak, Chip-Jin Ng, Chen-June Seak

**Affiliations:** ^1^Department of Emergency Medicine, Far Eastern Memorial Hospital, New Taipei City, Taiwan; ^2^School of Medicine, Institute of Emergency and Critical Care Medicine, National Yang Ming Chiao Tung University, Taipei, Taiwan; ^3^College of Medicine, Chang Gung University, Taoyuan, Taiwan; ^4^Department of Emergency Medicine, Lin-Kou Medical Center, Chang Gung Memorial Hospital, Taoyuan, Taiwan; ^5^Department of Chest Medicine, National Yang Ming Chiao Tung University Hospital, Yilan, Taiwan; ^6^Healthy Aging Research Center, Chang Gung University, Taoyuan, Taiwan; ^7^Laboratory for Epidemiology, Department of Health Care Management, Chang Gung University, Taoyuan, Taiwan; ^8^Research Center for Food and Cosmetic Safety, College of Human Ecology, Chang Gung University of Science and Technology, Taoyuan, Taiwan; ^9^Department of Safety, Health and Environmental Engineering, Ming Chi University of Technology, Taipei, Taiwan; ^10^Sarawak General Hospital, Kuching, Sarawak, Malaysia; ^11^Department of Emergency Medicine, New Taipei Municipal Tucheng Hospital, New Taipei City, Taiwan

**Keywords:** diaphragm, community-acquired pneumonia, respiratory failure, ultrasonography, point-of-care, stratification to prevent overcrowding taskforce (SPOT)

## Abstract

**Background:**

Early recognition of patients with community-acquired pneumonia (CAP) at risk of poor outcomes is crucial. However, there is no effective assessment tool for predicting the development of respiratory failure in patients with CAP. Diaphragmatic ultrasonography (DUS) is a novel technique developed for evaluating diaphragmatic function *via* measurements of the diaphragm thickening fraction (DTF) and diaphragm excursion (DE). This study evaluated the accuracy of DUS in predicting the development of respiratory failure in patients with CAP, as well as the feasibility of its use in the emergency department (ED) setting.

**Materials and methods:**

This was a single-center prospective cohort study. We invited all patients with ED aged ≥ 20 years who were diagnosed with CAP of pneumonia severity index (PSI) SIe diagnosed with CAP of pneumonia severe with respiratory failure or septic shock were excluded. Two emergency physicians performed DUS to obtain DTF and DE measurements. Data were collected to calculate PSI, CURB-65 score, and Infectious Diseases Society of America/American Thoracic Society severity criteria. Study endpoints were taken at the development of respiratory failure or 30 days post-ED presentation. Continuous variables were analyzed using *T*-tests, while categorical variables were analyzed using chi-square tests. Further logistic regression and receiver operating characteristic curve analyses were performed to examine the ability to predict the development of respiratory failure. Intra- and inter-rater reliability was examined with intraclass correlation coefficients (ICCs).

**Results:**

In this study, 13 of 50 patients with CAP enrolled developed respiratory failure. DTF was found to be an independent predictor (OR: 0.939, *p* = 0.0416). At the optimal cut-off point of 23.95%, DTF had 69.23% of sensitivity, 83.78% of specificity, 88.57% of negative predictive value, and 80% of accuracy. Intra- and inter-rater analysis demonstrated good consistency (intra-rater ICC 0.817, 0.789; inter-rater ICC 0.774, 0.781).

**Conclusion:**

DUS assessment of DTF may reliably predict the development of respiratory failure in patients with CAP presenting to the ED. Patients with DTF > 23.95% may be considered for outpatient management.

## Graphical Abstract

Diaphragmatic ultrasonography assessment of diaphragm thickening fraction (DTF) may reliably predict the development of respiratory failure in community-acquired pneumonia patients presenting to the emergency department. 
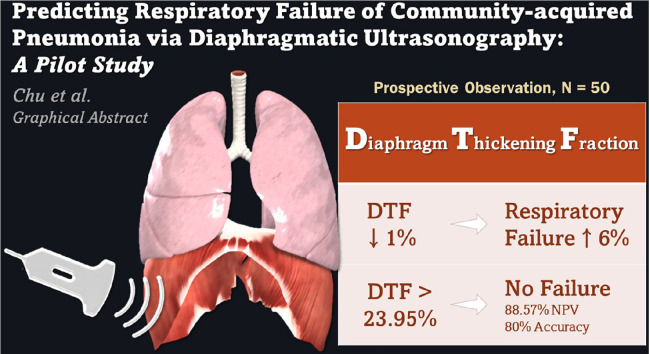

## Background

Pneumonia, an infectious disease of the pulmonary parenchyma, is a major cause of morbidity and mortality worldwide. It has been found to be the deadliest communicable disease and the 4^th^ leading cause of death globally in 2016, accounting for 3 million deaths (1, 2).

Community-acquired pneumonia (CAP) refers to pneumonia that is contracted by the patient in his own community environment, independently from healthcare facilities. Patients with CAP have a wide-ranging clinical presentation, varying from fever and cough to severe respiratory distress, acute respiratory distress syndrome, and even multiple organ dysfunction syndromes.

Early recognition of patients with the potential to deteriorate during their admission is important in the emergency department (ED) (3). To this end, various scoring tools have been evaluated in predicting the prognoses of patients with CAP, with the results being used to determine subsequent management (4–17). The pneumonia severity index (PSI) (7) has been recommended by the American Thoracic Society (ATS)/Infectious Diseases Society of America (IDSA) for use in the management of CAP (18), due to its well-validated, strong discriminatory power in risk stratification. PSI is, however, too complicated to calculate quickly and thus has limited clinical application in the ED. The CURB-65 score is another popular prediction tool (6) that is recommended by the British Thoracic Society and the National Institute for Health and Care Excellence (19). Despite its ease of use, the safety and effectiveness of CURB-65 in guiding clinical decision-making have not been determined.

Other scoring systems have been developed to identify patients with CAP who are critically ill and consequently require admission to the intensive care unit (ICU). These scores include the severe community-acquired pneumonia score (4), SMART-COP (16), and the IDSA/ATS major and minor severity criteria (17). These scoring systems are, however, not suitable for use in the ED, due to either their reliance on laboratory data that is not readily available in all ED settings or the time-consuming complex calculations required – these attributes would be a hindrance in the fast-paced ED where prompt decision-making is necessary.

Risk stratification *via* the abovementioned prognostic scores was performed by evaluating the risks of mortality of patients with CAP. Nevertheless, given that CAP imposes significant morbidity in addition to mortality, such risk assessment can also be performed through consideration of the morbidity risks of developing severe disease sequelae. One important complication of CAP would be respiratory failure, though there has yet to be any established evaluation method to predict its development in patients with CAP.

The diaphragm is a major inspiratory muscle and as such plays a critical role in the work of respiration (20). Diaphragmatic dysfunction has been proposed to be a marker of severe CAP, infection, and sepsis (21, 22). It is thought to represent a variant of organ failure (22) and is associated with failure to wean off mechanical ventilation as well as prolonged ICU stays (23).

Diagnosing diaphragmatic dysfunction was difficult and complicated previously (24–27), though the advent of point-of-care ultrasonography has led to huge strides in this aspect (28–31). The diaphragmatic function can currently be evaluated *via* 2 main ultrasonography techniques. The first technique, diaphragm thickening fraction (DTF), is a measurement of the difference in end-inspiratory and end-expiratory diaphragmatic thickness, expressed as a fraction with the denominator of the end-expiratory thickness. The second method is to measure diaphragm excursion (DE), the diaphragmatic altitude difference between expiration and inspiration (20).

Both DTF and DE were originally developed for use in the ICU to assess patients’ suitability to be weaned off mechanical ventilation. A few studies have assessed the practicality and effectiveness of diaphragmatic ultrasonography (DUS) outside the ICU, demonstrating good intra- and inter-rater reproducibility of right-sided DE measurements in the ED (32–34). Its utility in predicting the need for mechanical ventilation has, however, not been fully examined.

In this study, we aimed to evaluate the two DUS techniques of DTF and DE in predicting the development of respiratory failure in patients with CAP presenting to the ED. These results will assist emergency physicians (EPs) and intensivists in the early identification of patients with CAP at risk of deteriorating rapidly and subsequently enable clinicians to make necessary arrangements in advance for mechanical ventilation and ICU admission.

## Materials and methods

### Study design and setting of the study

This was a single-center, prospective cohort study of all patients who visited the ED of Linkou Chang Gung Memorial hospital from December 2018 to March 2019 and were diagnosed with CAP. Linkou Chang Gung Memorial Hospital is one of the largest tertiary medical centers in the world with 3,406 beds and approximately 15,000 monthly ED visits in 2019 (35). This study was approved by the Institutional Review Board of the Chang Gung Memorial Hospital (IRB no: 201801855B0).

### Patient recruitment

All patients aged 20 years or older who visited our ED and were diagnosed with CAP of moderate-to-high severity (PSI class ≥ 4) were invited to participate in this study, except for those who fulfilled the following exclusion criteria: [1] history of prior admission to any healthcare facility in the preceding 48 h; [2] patients who meet the IDSA/ATS severity major criteria (17) on ED presentation, i.e., already in respiratory failure requiring immediate mechanical ventilation or in septic shock requiring vasopressor support; or [3] patients visiting the ED more than once with the same diagnosis. Eligible participants were counseled regarding the objectives and details of our study prior to informed consent being obtained.

### Management protocol of community-acquired pneumonia

All patients diagnosed with CAP in our ED were managed by EP-led teams in accordance with the 2018 Taiwanese guidelines, endorsed by the Infectious Diseases Society of Taiwan and the Taiwan Society of Pulmonary and Critical Care Medicine (36). Appropriate initial treatments were commenced in line with our hospital’s standard treatment protocols approved by the hospital’s ED committee, based on each patient’s history, initial clinical evaluation, and vital signs on presentation. PSI, CURB-65 scores, and IDSA/ATS severity criteria were assessed to assist the attending EP in deciding the need for hospital admission and/or intensive care.

### Patient evaluation *via* diaphragmatic ultrasonography

After eligible patients had consented to participate in our study, DUS was performed immediately by two board-certified EPs (raters A and B) who had been trained in emergency ultrasonography and are accredited by both the Taiwan Society of Emergency Medicine and the Taiwan Society of Ultrasound in Medicine. These two raters were blinded to the patients’ full diagnoses and treatment courses, while patients and attending clinical teams were blinded to the ultrasonography findings.

Diaphragmatic ultrasonography was performed at the bedside using the Mindray M7 portable ultrasound machine. Each examination comprised measurements, which were repeated for three consecutive respiratory cycles and subsequently averaged. Both raters took turns performing the DUS examination twice on the right hemidiaphragm. The right hemidiaphragm was opted over the left hemidiaphragm since the latter was often obscured by gastric contents with a less-favorable spleen window (28, 32, 37). All DTF and DE measurements obtained were recorded accordingly for statistical analysis.

The clinical progress of all recruited patients was followed. Study endpoints were taken at either development of respiratory failure or 30 days post-ED presentation.

### Technique to measure diaphragm thickening fraction

The diaphragm thickening fraction is calculated using the following formula:


(1) $$D\,T\,F=\frac{\begin{array}{c} \left(e\,n\,d-i\,n\,s\,p\,i\,r\,a\,t\,o\,r\,y\,d\,i\,a\,p\,h\,r\,a\,g\,m\,t\,h\,i\,c\,k\,n\,e\,s\,s\right)\\ -\left(e\,n\,d-e\,x\,p\,i\,r\,a\,t\,o\,r\,y\,d\,i\,a\,p\,h\,r\,a\,g\,m\,t\,h\,i\,c\,k\,n\,e\,s\,s\right) \end{array}}{e\,n\,d-e\,x\,p\,i\,r\,a\,t\,o\,r\,y\,d\,i\,a\,p\,h\,r\,a\,g\,m\,t\,h\,i\,c\,k\,n\,e\,s\,s}$$

**FIGURE 1:**
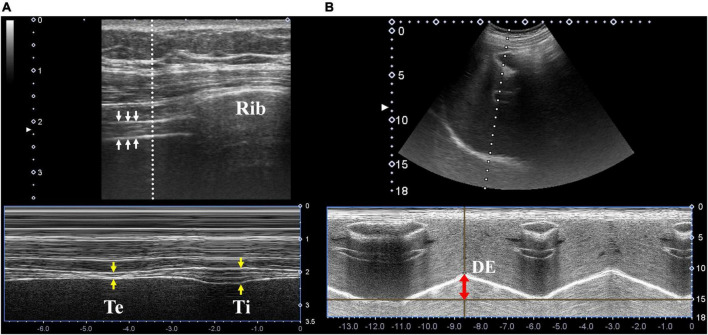
Diaphragmatic ultrasonography. **(A)** Ultrasonographic images of the diaphragm (white arrows) in the longitudinal plane were located in the right 7^th^ intercostal space along the mid-axillary line. The thickness at end expiration (yellow arrows, labeled as “Te”) and end inspiration (labeled as “Ti”) were measured using M-mode ultrasonography. **(B)** Ultrasonographic images of the diaphragm in the sagittal plane were located in the right subcostal area between the mid-clavicular and anterior axillary lines. Diaphragm excursion was measured using M-mode ultrasonography (red double-head arrow).

It was assessed *via* ultrasound through the 7^th^ to 9^th^ intercostal spaces at the right mid-axillary line using M-mode while the patients lay in the supine position. The evaluation was performed using a high-frequency linear probe (Mindray L12-4s probe, 6–10 MHz) in the longitudinal plane (Figure 1A).

### Technique to measure diaphragm excursion

Diaphragm excursion was assessed using the right subcostal view (below the right costal margin between mid-clavicular and anterior axillary lines) while the patients lay in the supine position. The evaluation was performed using a low-frequency linear probe (Mindray C5-2s probe, 2.5–6 MHz) in the sagittal plane, with cranial angulation to produce a perpendicular cross-sectional image of the posterior diaphragm using the liver as an acoustic window (Figure 1B).

### Data analysis

Continuous variables were recorded as means ± standard deviation and analyzed using *T*-tests, while categorical variables were expressed as frequencies with their corresponding percentages and evaluated *via* chi-square tests. The averages of the 4 DTF and 4 DE measurements recorded for each patient with CAP were used for statistical analysis between patients who developed respiratory failure vs. those who did not. Logistic regression models were also constructed to assess the association between the variables and study outcomes. Receiver operating characteristic (ROC) curve analysis with c-statistics was performed, with optimal cut-off points identified for DTF and DE. Areas under the ROC curve (AUROC) were plotted for DTF, DE, PSI, CURB-65, and the IDSA/ATF minor criteria to compare their abilities to predict the development of respiratory failure in patients with CAP.

To assess the intra- and inter-rater reliability of DUS, intraclass correlation coefficients (ICCs) with 95% confidence intervals were calculated, based on a mean-rating (*k* = 2), single-measure, and 2-way mixed effects model (38). All statistical analyses were performed using Statistical Analysis Software version 9.4 and Statistical Package for the Social Sciences version 24.0. Statistical significance was taken at 0.05.

## Results

In this study, 50 patients with CAP who presented to the ED from December 2018 to March 2019 were recruited for this study. *Post-hoc* analysis found this sample size to be sufficiently powered at 86.9%. These patients had a mean age of 78 years, with a majority of them being men (56%). Notably, 13 of these 50 patients with CAP (26%) eventually developed respiratory failure during hospitalization, approximately 7 days after presenting to the ED (165.6 ± 143.3 h). In comparison with patients who did not develop respiratory failure, patients with CAP who progressed into respiratory failure were found to be more anemic (*p* = 0.0087) with higher blood urea nitrogen levels (*p* = 0.0264). There were no statistically significant differences in patient demographics, underlying comorbidities, initial vital signs, and other laboratory investigation results between both groups (Table 1).

**TABLE 1:** Characteristics of community-acquired pneumonia (CAP) patients who developed respiratory failure versus those who did not.

	All patients	Respiratory failure	No respiratory failure	*P*-value
Total number of patients	50	13	37	
Age (years)	78 ± 11.85	76.69 ± 12.04	78.46 ± 11.91	0.6484
Male	28 (56)	8 (61.54)	20 (54.02)	0.8864
Time from symptoms onset to ED arrival (days)	2.44 ± 1.99	3.23 ± 2.01	2.16 ± 1.94	0.0964
Time from ED arrival to DUS (hours)	11.41 ± 7.31	8.95 ± 6.17	12.3 ± 7.56	0.1586
Time from ED arrival to respiratory failure (hours)	–	165.6 ± 143.33	–	–
**Vital Sign at triage**				
Body Temperature (°C)	36.91 ± 1.56	36.57 ± 1.55	37.03 ± 1.56	0.3671
Heart Rate (/min)	102.72 ± 23.42	102.7 ± 22.22	102.7 ± 24.12	0.9961
Respiratory Rate (/min)	24.3 ± 5.37	23.69 ± 5.3	24.51 ± 5.45	0.64
Systolic Blood Pressure (mmHg)	129.32 ± 31.94	118.9 ± 28.76	133 ± 32.55	0.1749
Diastolic Blood Pressure (mmHg)	74.1 ± 17.88	70.31 ± 16.48	75.43 ± 18.38	0.3796
Glasgow Coma Scale	12.88 ± 3.05	13 ± 3.16	12.84 ± 3.05	0.8709
Altered mental status	11 (22)	3 (23.08)	8 (21.62)	1
**Initial Blood test**				
Leucocyte count (× 10^6^/L)	11904 ± 6106.03	10476.9 ± 5453.6	12405.4 ± 6311.8	0.3324
Segmented cells (%)	79.61 ± 14.87	73.17 ± 24.08	81.88 ± 9.34	0.2256
Band form cells (%)	0.62 ± 1.46	1.08 ± 1.61	0.46 ± 1.39	0.1911
Lymphocytes (%)	10.31 ± 6.81	12.06 ± 9.87	9.68 ± 5.36	0.4221
Hemoglobin (g/dL)	**11.4 ± 2.04**	**10.15 ± 2.13**	**11.84 ± 1.84**	**0.0087**
Haematocrit (%)	**34.48 ± 6.24**	**30.88 ± 6.97**	**35.74 ± 5.53**	**0.0142**
C-reactive protein	99.16 ± 90.49	139.2 ± 113.5	85.01 ± 77.96	0.0739
Blood urea nitrogen	**33.28 ± 29.24**	**54.89 ± 40.82**	**25.68 ± 19.49**	**0.0264**
Creatinine (μmol/L)	1.76 ± 1.76	2.59 ± 2.4	1.46 ± 1.4	0.1306
Sodium (mmol/L)	134.96 ± 6.36	137.4 ± 9.27	134.1 ± 4.85	0.2431
Potassium (mmol/L)	3.96 ± 0.63	4.25 ± 0.79	3.85 ± 0.54	0.0530
pH	7.35 ± 0.47	7.41 ± 0.06	7.33 ± 0.55	0.4098
HCO_3_ (mmol/L)	24.06 ± 5.07	23.56 ± 5.87	24.25 ± 4.82	0.6939
pCO_2_ (mmHg)	36.72 ± 9.21	37.12 ± 7.23	36.56 ± 9.98	0.8626
**Comorbidity**				
From nursing home	5 (10)	1 (7.69)	4 (10.81)	1
Active cancer	18 (36)	6 (46.15)	12 (32.43)	0.5042
Congestive heart failure	8 (16)	2 (15.38)	6 (16.22)	1
Diabetes mellitus	19 (38)	7 (53.85)	12 (32.43)	0.1991
Cerebrovascular disease	22 (44)	5 (38.46)	17 (45.95)	0.8864
Dementia	6 (12)	1 (7.69)	5 (13.51)	1
Chronic pulmonary disease	16 (32)	3 (23.08)	13 (35.41)	0.5075
Peptic ulcer disease	2 (4)	0 (0)	2 (5.41)	1
Chronic liver disease	2 (4)	2 (15.38)	0 (0)	0.0637
Paraplegia and hemiplegia	6 (12)	1 (7.69)	5 (13.51)	1
Chronic renal disease	7 (14)	2 (15.38)	5 (13.51)	1
**Risk scores**				
PSI	**123 ± 26.10**	**146.3 ± 26.30**	**114.8 ± 20.87**	** < 0.0001**
CURB-65	1.92 ± 0.94	2.31 ± 1.11	1.78 ± 0.85	0.0852
IDSA/ATS minor criteria	**1.4 ± 1.09**	**2.15 ± 1.21**	**1.14 ± 0.92**	**0.0185**
**Diaphragm ultrasound**				
Diaphragm excursion (cm)	1.91 ± 0.72	1.62 ± 0.53	2.01 ± 0.76	0.092
Diaphragm thickening fraction	**0.37 ± 0.18**	**0.26 ± 0.15**	**0.41 ± 0.17**	**0.0094**

ED, emergency department; DUS, diaphragmatic ultrasonography; PSI, pneumonia severity index; IDSA/ATS, Diseases Society of America/American Thoracic Society.

In terms of risk stratification tools assessed in the ED, patients with CAP who developed respiratory failure were found to have higher PSI scores (*p* < 0.0001) and fulfilled more IDSA/ATS minor criteria (*p* = 0.0185). DUS examination revealed that DTF in patients who developed respiratory failure was lower at 26 ± 15% vs. 41 ± 17% in those who did not (*p* = 0.0094), while there were no significant differences in DE measurements for both groups (Table 1).

Further logistic regression analyses were performed for each risk stratification tool. Univariate analysis found that DTF (OR 0.934, *p* = 0.0178), PSI (OR 1.050, *p* = 0.0012), and IDSA/ATS minor criteria (OR 2.585, *p* = 0.0078) had significant correlation with the development of respiratory failure. Subsequent multivariate analysis revealed that only DTF and PSI were independent predictors. Each reduction of 1% in DTF increased the odds of developing respiratory failure by 6.1% (Table 2).

**TABLE 2:** Logistic regression of risk stratification tools for predicting respiratory failure in patients with community-acquired pneumonia.

	Univariate analysis		Multivariable analysis	
Predictor	Odd ratio	*P*-value	Odd ratio	*P*-value
DTF (every 1% increase)	0.934	0.0178	0.939	0.0416
DE	0.392	0.1006	–	–
PSI	1.050	0.0012	1.063	0.0314
CURB-65	1.863	0.0914	–	–
IDSA/ATS minor criteria	2.585	0.0078	1.021	0.9759

DTF, diaphragm thickening fraction; DE, diaphragm excursion; PSI, pneumonia severity index; IDSA/ATS, Diseases Society of America/American Thoracic Society.

**FIGURE 2:**
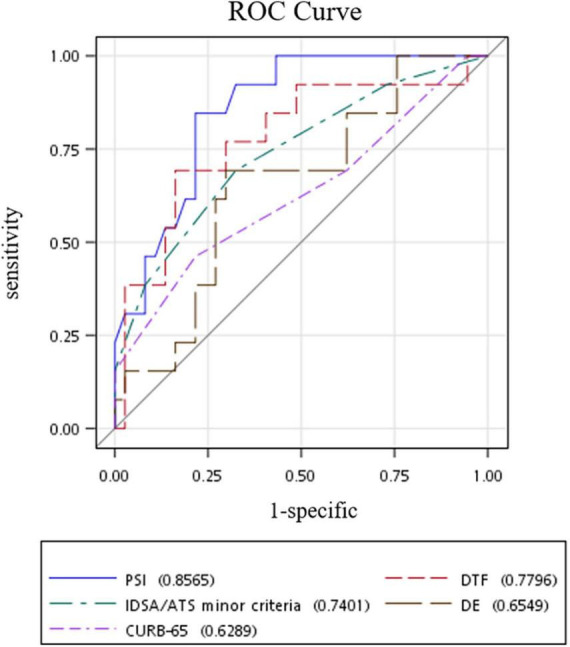
ROC curves of risk stratification tools in predicting respiratory failure in patients with community-acquired pneumonia. ROC, receiver operating characteristic; DTF, diaphragm thickening fraction; DE, diaphragm excursion; PSI, pneumonia severity index; IDSA/ATS, Diseases Society of America/American Thoracic Society.

Receiver operating characteristic (ROC) curve analysis of the studied risk stratification tools found PSI to be the best performing tool with an area under ROC curve (AUROC) of 0.8565, while DTF ranked second with an AUROC of 0.7796 (Figure 2). Further comparison of both ROC curves revealed that both PSI and DTF were comparable in their abilities to predict the development of respiratory failure (Pr > ChiSq = 0.3841) (Table 3). The optimal cut-off point, identified *via* maximizing Youden’s index, was found to be 23.95% for DTF, with a corresponding sensitivity of 69.23%, specificity of 83.78%, negative predictive value of 88.57%, and accuracy of 80% (Table 4).

**TABLE 3:** Area under the receiver operating characteristic curve (AUROC) analysis of risk stratification tools in predicting development of respiratory failure.

	AUROC	95% CI	
DTF	0.7796	(0.6184, 0.9408)	
DE	0.6549	(0.4835,0.8262)	
PSI	0.8565	(0.7524,0.9606)	
CURB-65	0.6289	(0.4406,0.8172)	
IDSA/ATS minor criteria	0.7401	(0.5787,0.9016)	
**Comparison of AUROC**			
**Contrast**	**Difference between areas**	**95% CI**	**Pr > ChiSq**
DTF – PSI	–0.1025	(–0.3333, 0.1283)	0.3841
DTF – IDSA/ATS minor criteria	0.02	(–0.2616, 0.3016)	0.8893

AUROC, Area under the receiver operating characteristic curve; DTF, diaphragm thickening fraction; DE, diaphragm excursion; PSI, pneumonia severity index; IDSA/ATS, Diseases Society of America/American Thoracic Society; 95% CI: 95% confidence interval; Pr > ChiSq: probability > chi-square.

**TABLE 4:** Optimal cut-off point for diaphragm thickening fraction (DTF) with its corresponding sensitivity, specificity, and accuracy.

Cut-off point	Sen	Sp	Acc	PPV	NPV	LR+	LR-
23.95%	69.23%	83.78%	80%	60%	88.57%	4.2692	0.3672

DTF, diaphragm thickening fraction; Sen, sensitivity; Sp, specificity; Acc, accuracy; PPV, positive predictive value; NPV, negative predictive value; LR+, positive likelihood ratio; LR–, negative likelihood ratio.

Evaluation of DTF’s intra- and inter-rater reliabilities demonstrated good consistency, with 0.774 (95% CI: 0.633 – 0.865) and 0.781 (95% CI: 0.644 – 0.870) inter-rater intraclass correlation coefficients (ICCs) and 0.817 (95% CI: 0.700 – 0.892) and 0.789 (95% CI: 0.657 – 0.874) intra-rater ICCs (Table 5).

**TABLE 5:** Reliability of the diaphragm thickening fraction (DTF) in emergency department.

		ICC	95% CI
Inter-rater reliability	1st DTF	0.774	(0.633,0.865)
	2nd DTF	0.781	(0.644,0.870)
Intra-rater reliability	Rater A	0.817	(0.700,0.892)
	Rater B	0.789	(0.657,0.874)

DTF, diaphragm thickening fraction; ICC, intraclass correlation coefficient; 95% CI, 95% confidence interval.

## Discussion

Our study demonstrates that the DTF technique of DUS can reliably predict the development of respiratory failure in patients with CAP presenting to the ED. We also found that DE is a poor predictor of the development of respiratory failure. This study is, to the best of our knowledge, the first to describe the association of critical illness-associated diaphragm dysfunction with the development of respiratory failure in patients with CAP.

Early identification of patients with CAP at risk of deteriorating rapidly *via* point-of-care ultrasonography would enable clinicians to make necessary arrangements in advance for mechanical ventilation and ICU admission. This prognostication and risk stratification tool may also allow clinicians to prioritize and optimize the care of these critically ill patients with CAP and facilitate counseling and communication with patients and their families from the very beginning of their hospital admissions. Conversely, patients with a low risk of developing respiratory failure can be considered for outpatient management.

During the respiratory cycle, the diaphragm thickens in the inspiratory phase while moving caudally to create negative intrathoracic pressure for drawing air into the lungs; conversely, the diaphragm flattens out during expiration while moving cranially in the expiratory phase to create a positive intrathoracic pressure to expel air out of the lungs. Diaphragmatic weakness is highly prevalent in critically ill patients. Often referred to as “ventilator-induced diaphragmatic dysfunction,” it is a well-recognized pathological entity in the ICU (39). Recent findings of its occurrence even before ICU admission have led to the coining of a new term – “critical illness-associated diaphragm weakness” (40). The DTF is a measurement of the changing diaphragmatic thickness during respiration and, therefore, reflects the magnitude of active diaphragmatic contraction (41). The lower the DTF, the poorer the diaphragmatic contractility, i.e., the greater the extent of diaphragmatic dysfunction.

This direct representation of the diaphragm’s function is perhaps the reason why we found a strong correlation between DTF and the development of respiratory failure in patients with CAP presenting to the ED. Since it provides a preview of diaphragmatic dysfunction, DTF may be used to enable early risk stratification and rapid decision-making at the bedside in the ED – future follow-up studies can be performed to evaluate and validate its clinical application.

Stroke, neuromuscular disease, age, and cardiopulmonary diseases such as heart failure and chronic obstructive pulmonary disease have been associated with diaphragmatic dysfunction (24, 42–46). This is most probably due to the poor physiological reserves of patients with these comorbidities, manifesting as decreased respiratory function and correspondingly decreased DTFs. The significant correlation between DTF and development of respiratory failure in our study is, however, unlikely to be accounted for by this phenomenon, as comparisons of underlying comorbidities between both patient groups found no statistically significant differences (Table 1). A probable explanation is that the patients with these comorbidities severe enough to cause an impaired respiratory function at their baselines would have presented in a bad enough shape to have fulfilled our exclusion criteria and, consequently, were not included in this study. Further studies can consider collecting data on such patients with CAP who are already in respiratory failure on ED presentation to test this hypothesis.

While the DTF was found to be a valuable risk stratification tool in predicting the development of respiratory failure in patients with CAP at the ED, the same does not hold true for the DE. This is perhaps because DTF directly evaluates the extent of diaphragmatic contractility (and therefore function) by measuring the variability of its thickness during the respiratory cycle, while DE is an indirect evaluation of this contractility by measuring the altitude displacement of the diaphragm between inspiration and expiration. Not only is this DE measurement of altitude displacement influenced by active diaphragmatic contractions, it is also affected by other forces external to the diaphragm (41). During inspiration, the negative pressure generated by the accessory inspiratory muscles pulls the diaphragm cranially; conversely, the positive pressure generated by the accessory expiratory muscles during expiration pushes the diaphragm cranially. When there is diaphragmatic dysfunction, these accessory muscles all work in conjunction to compensate for the weak diaphragm to maintain a similar respiratory volume and therefore resulting in a similar DE on DUS, albeit with a paradoxical pattern (41, 47). Our finding that DE is a poor predictor of the development of respiratory failure echoes those of previous studies (32, 33).

Univariate logistic regression analysis of the various risk stratification tools found that PSI, IDSA/ATS minor criteria, and DTF were significantly associated with the development of respiratory failure in patients with CAP, while multivariate analysis confirmed that PSI and DTF were independent predictors. Further AUROC analysis revealed that PSI and DTF were comparable in their risk stratification abilities. While statistical analysis had demonstrated that DTF was noninferior to the more established PSI, the former is more clinically feasible and applicable in the ED environment. This is especially so with the increasing ubiquity of point-of-care ultrasonography in the ED, which would allow rapid visualization of the diaphragm and DTF measurements within minutes. In stark contrast, calculating the PSI would require a much longer time to collate the required data on the patient’s comorbidities, clinical history, physical examination, laboratory investigation results, and radiological imaging.

At a cut-off point of 23.95%, DTF was found to be 80% accurate in predicting the development of respiratory failure in patients with CAP, with a sensitivity of 69.23% and a specificity of 83.78%. Its high NPV of 88.57% and negative likelihood ratio of 0.3672 mean that patients with DTF > 23.95% are very unlikely to develop respiratory failure; these patients can be considered for outpatient management and follow-up. Patients with DTF < 23.95% have a high likelihood of developing respiratory failure (positive likelihood ratio 4.2692) and therefore would benefit from hospitalization and monitoring by healthcare professionals, especially in the first week of illness (mean time from ED arrival to respiratory failure of 165 h).

Our study is a single-center observational study with a relatively small sample size. Therefore, further studies should be performed to confirm our findings and validate the use of DUS and DTF assessment in patients with CAP. As with any other ultrasonography-based evaluation, DUS is operator-dependent (48). Nevertheless, our study demonstrated high intra- and inter-rater reliabilities when DUS was performed by properly trained operators well-versed in ultrasonography. Future studies can be performed to evaluate the feasibility of this assessment modality in the hands of junior doctors with less experience, to determine if adequate intra- and inter-rater reliability can still be maintained after proper training in line with the EXODUS consensus statement (49, 50).

## Conclusion

Diaphragmatic ultrasonography assessment of DTF in patients with CAP may be a reliable and accurate risk stratification tool to predict the development of respiratory failure. The short time required by a trained operator to obtain these measurements makes it ideal for the ED setting. Patients with DTF > 23.95% may be considered for outpatient management – further studies are, however, required to validate its use in the clinical management of CAP in the ED.

## Members of the SPOT investigators

The SPOT includes the following members: Johan Seak, Jia-Qi Xu, Yi-Zhen Chen, Hao-Tsai Cheng, Hsien-Yi Chen, Yu-Shao Chou, Chih-Huang Li, Tzu-Heng Cheng, Chen-Bin Chen, Chia-Hau Chang, Chien-Lin Chen, Wei-Chun Lin, and Chiao-Hsuan Hsieh.

## Data availability statement

The datasets generated and/or analyzed during this current study are available from the corresponding author on reasonable request.

## Ethics statement

The studies involving human participants were reviewed and approved by Institutional Review Board of the Chang Gung Memorial Hospital (IRB no: 201801855B0). The patients/participants provided their written informed consent to participate in this study. Written informed consent was obtained from the individual(s) for the publication of any potentially identifiable images or data included in this article.

## Author contributions

K-HH: approve the formal analysis. S-EC, J-XL, and C-JS: conceptualization. S-EC and J-XL: data curation. K-HH and C-JS: formal analysis and supervision. C-JS: funding acquisition and resources. S-CC, ZG, C-KS, JS, and C-JS: methodology. S-EC, J-XL, C-JN, and C-JS: investigation. C-JS and ZG: validation, visualization, and writing – review and editing. S-EC, J-XL, ZG, C-KS, JS, and C-JS: writing – original draft. All authors contributed to the article and approved the submitted version.

## Abbreviations

CAP: community-acquired pneumonia
CI: confidence interval
DUS: diaphragmatic ultrasonography
DTF: diaphragm thickening fraction
DE: diaphragm excursion
ED: emergency department
PSI: pneumonia severity index
ATS: American Thoracic Society
IDSA: Infectious Diseases Society of America
ICU: intensive care unit
EP: emergency physician
ROC: receiver operating characteristic
AUROC: area under the receiver operating characteristic
RF: respiratory failure
ICC: intraclass correlation coefficients.
